# Prediction of peak pressure from clinical and radiological measurements in patients with diabetes

**DOI:** 10.1186/1472-6823-8-16

**Published:** 2008-12-02

**Authors:** Nick A Guldemond, Pieter Leffers, Geert HIM Walenkamp, Nicolaas C Schaper, Antal P Sanders, Fred HM Nieman, Lodewijk W van Rhijn

**Affiliations:** 1 Department of Orthopaedic Surgery, University Hospital Maastricht, the Netherlands; 2 Department of Epidemiology, University Maastricht, the Netherlands; 3 Department of Rehabilitation Medicine, University Hospital Maastricht, the Netherlands; 4 Department of Internal Medicine, University Hospital Maastricht, the Netherlands; 5 Department of Clinical Epidemiology and Medical Technology Assessment, University Hospital Maastricht, the Netherlands

## Abstract

**Background:**

Various structural and functional factors of foot function have been associated with high local plantar pressures. The therapist focuses on these features which are thought to be responsible for plantar ulceration in patients with diabetes. Risk assessment of the diabetic foot would be made easier if locally elevated plantar pressure could be indicated with a minimum set of clinical measures.

**Methods:**

Ninety three patients were evaluated through vascular, orthopaedic, neurological and radiological assessment. A pressure platform was used to quantify the barefoot peak pressure for six forefoot regions: big toe (BT) and metatarsals one (MT-1) to five (MT-5). Stepwise regression modelling was performed to determine which set of the clinical and radiological measures explained most variability in local barefoot plantar peak pressure in each of the six forefoot regions. Comprehensive models were computed with independent variables from the clinical and radiological measurements. The difference between the actual plantar pressure and the predicted value was examined through Bland-Altman analysis.

**Results:**

Forefoot pressures were significant higher in patients with neuropathy, compared to patients without neuropathy for the whole forefoot, the MT-1 region and the MT-5 region (respectively 138 kPa, 173 kPa and 88 kPa higher: mean difference). The clinical models explained up to 39 percent of the variance in local peak pressures. Callus formation and toe deformity were identified as relevant clinical predictors for all forefoot regions. Regression models with radiological variables explained about 26 percent of the variance in local peak pressures. For most regions the combination of clinical and radiological variables resulted in a higher explained variance. The Bland and Altman analysis showed a major discrepancy between the predicted and the actual peak pressure values.

**Conclusion:**

At best, clinical and radiological measurements could only explain about 34 percent of the variance in local barefoot peak pressure in this population of diabetic patients. The prediction models constructed with linear regression are not useful in clinical practice because of considerable underestimation of high plantar pressure values. Identification of elevated plantar pressure without equipment for quantification of plantar pressure is inadequate. The use of quantitative plantar pressure measurement for diabetic foot screening is therefore advocated.

## Introduction

Clinical examination is considered important in the evaluation of the diabetic foot. Physicians and foot care specialists should be able to identify patients at risk of adverse outcomes such as ulceration. A structured clinical assessment that incorporates diagnostic tests alongside a thorough history and examination is thought to be essential for preventive strategies[1]. High plantar pressures have been shown to be related to development of foot ulcers in people with diabetes. However, no study has assessed the relative importance of clinical examination in relation to the prediction of peak pressure.

Various structural and functional factors of foot function have been associated with high local plantar pressures such as limited joint mobility [2-4], plantar soft tissue thickness, stiffness and callosities [5-17], metatarsal length [18-20], the configuration of the medial longitudinal arch [21-24], the presence of metatarsal deformities[25,26] and toe deformities[27,28]. These features are associated with locally elevated plantar pressure which could cause plantar ulceration in patients with diabetes. To decide whether a diabetic foot is at risk, foot care specialists make an inventory of these features through observation, physical examination and radiographic evaluation. Risk assessment of the diabetic foot would be made easier if locally elevated peak pressure could be indicated with a minimum set of commonly used clinical measures.

An alternative diagnostic screening method for abnormal physical stress on the foot sole is direct quantitative measurement of plantar pressure. In the last few decades electronic devices such as pressure sensitive platforms have become commercially available for this purpose. This equipment is relatively expensive and there is no reimbursement by health insurers of the costs for the provision of this screening procedure. Consequently, this diagnostic facility is seldom provided as standard care in clinical settings.

The primary objective of this study was to assess the relationship between clinical measurements, radiological data and barefoot plantar pressure in order to find clinical measures that predict local peak pressure in patients with diabetes. In addition, we also evaluated previously proposed regression models by various authors with the same and/or similar variables as those obtained in our current study. To evaluate the effect of peripheral neuropathy as a predictor in this study, we included both patients with – and without peripheral neuropathy. The secondary objective of this study was to assess the differences in clinical measurements, radiological data and barefoot peak pressure between patients with – and without peripheral neuropathy.

## Methods

### Patients

Diabetic patients were selected from the outpatient clinic of the University Hospital Maastricht. Inclusion criteria were diabetes mellitus type 1 (longer than 10 years after date of diagnosis) or type 2 (at least one year after date of diagnosis); age between 30 and 75 years and able to perform daily-life activities without supporting devices. Exclusion criteria were a history of rheumatoid arthritis, severe foot trauma, severe deformity i.e. which require orthopaedic shoes and/or surgery of the foot.

Before the start of the study, patients were informed about all study procedures and their possible risks. The Research Ethical Committee of the University Hospital Maastricht approved the study. One hundred and twenty five eligible patients were screened for peripheral neuropathy through determination of the vibration perception threshold (VPT) at the apex of the hallux with a biothesiometer (Biomedical, Newbury OH)[29,30]. A VPT higher than 25 Volts was used as the diagnostic criterion for peripheral neuropathy. Table 1 contains descriptive data with respect to gender, age, body mass index (BMI), duration of diabetes and glycosylated haemoglobin (HBa1c). Thirty two patients were excluded because of corrupted plantar pressure data due to technical failure. Finally, data of 93 patients were used for analysis. None of the subjects had a history of foot ulceration.

**Table 1:** Patient characteristics

	**PNP-**						**PNP+**					
n	**49**						**44**					
Gender (female/male)	**30/19**						**29/15**					
Type of DM (1/2)	**18/31**						**9/35**					
	**female**			**male**			**female**			**male**		
	**Mean**	**SD**	**Range**	**Mean**	**SD**	**Range**	**Mean**	**SD**	**Range**	**Mean**	**SD**	**Range**

Age (yr)	56.3	9.1	35	50.9	9.5	38	64.9	8.8	26	58.8	10.4	44
Duration diabetes (yr)	13.8	10.2	36	11.6	7.7	31	18.1	11.4	38	16.3	11.4	42
HBa1c (percent)	8.31	1.5	4.8	8.15	1.1	46	8.25	1.3	5.5	8.16	1.3	6.3
BMI (kg/m^2^)	28.9	5.2	19.8	28.2	4.8	17.9	31.2	7.4	25.7	29.6	6.5	27.3

VPT = vibration perception threshold, PNP+ = peripheral polyneuropathy (VTP > 25 Volts), PNP- = no peripheral polyneuropathy (VPT ≤ 25 Volts), DM = diabetes mellitus, HbA1c = hemoglobin A1c, yr = year, BMI = Body Mass Index

### Barefoot plantar pressure measurement

An EMED SF-4^® ^pressure sensitive platform (Novel, Munich) was used to quantify the barefoot plantar pressures of the patients' feet and was performed according to a one-step protocol[31,32]. Barefoot peak pressure was estimated per foot by calculating the mean over the readings of 5 steps. This was done for the whole forefoot and six separate forefoot regions: big toe and metatarsals one (MT-1) to five (MT-5) through weight-bearing anterior-posterior radiographs and Novel 'clinics^®^' software[33].

Prior to data collection the grade of callus formation under the plantar aspect of the hallux and the metatarsals' heads was scored according to the method described by Colagiuri et al[17] (1995). The definitions of scores were: grade 1) Distinct area with minimal thickening of keratin layer, grade 2) moderate thickening of keratin layer, grade 3) marked thickening of keratin layer. The callus formation was not removed prior to the data collection, because this was a factor of interest in the prediction of peak pressure.

### Peripheral neurological assessment

The 'Valk' scoring system[34] for clinical neurological examination was performed to assess the grade of polyneuropathy. Pinprick sense and light touch sense (cotton wool) of the dorsum of the foot was tested on the mid-foot and compared to the proximal quality of sensation of the ankle. The quality of the vibration sense was tested with a 128 Hz tuning fork. The vibration sense of the big toe was compared to the vibration sense of the ankle, and the vibration sense of the ankle was compared to the patella. The ankle reflex action was compared to the knee reflex action. Pinprick sense, light touch sense, vibration sense of toes and ankles, and ankle reflexes were separately scored for both feet. Criteria for scoring for: normal (0), impaired in comparison with proximal (1) and absent (2), summing up to a maximum score of 20 points. Additionally, light touch sense was related to the anatomical level below which it was impaired: no abnormalities (0), toe (1), mid-foot (2), ankle (3), mid-calf (4) and knee (5). The outcome of the total score could vary between 0 and 25. According to Valk et al (1997) a score higher than 4 was graded as peripheral polyneuropathy[35].

### Orthopaedic assessment

The passive ankle joint dorsal flexion range of motion was measured through use of a plastic goniometer with the patient in prone position and the knee flexed[36]. The range of dorsiflexion of the first metatarsophalangeal joint relative to the first metatarsal shaft was measured in a non-weight bearing position. The bisection lines of the goniometer were placed along the medial shaft of the first metatarsal and the proximal phalanx of the hallux. The degree of hallux valgus was measured in accordance with the guidelines of the American Academy of Orthopaedic Surgeons[37].

The presence of claw toe, mallet toe, curly toe or hammer toe deformity was scored according to the definitions described by Myerson and Shereff[27]. These definitions were used as references for classification of the degree of toe deformity. Accordingly, toe alignment could be normal or deformed as defined by Myerson and Shereff: i.e. 'normal toe' was recorded if there was no deformity present. In addition, less and more malalignment than the definitions described by Myerson and Shereff was arbitrarily classified as minor and severe.

From a dorsal view, a crude judgement of the resting calcaneal stance position was made through visual inspection while the patient was in standing at rest position. The resting calcaneal stance position was recorded as neutral, varus or valgus alignment. In addition, the medial arch height was subjectively classified into: no arch (pes planus); lowered arch; normal arch; elevated arch and excessively elevated arch.

### Vascular assessment

For vascular tests, the patient lay supine and rested for at least 15 minutes prior to testing. A standard examination protocol for each lower extremity was performed. Resting ankle-brachial index (ABI) was obtained through continuous wave Doppler technique and performed at a 45 degree angle to the skin. Ankle-brachial index was calculated with the highest Doppler derived systolic pressure at the ankle (dorsalis pedis or posterior tibialis artery), indexed to the highest Doppler derived brachial artery pressure.

A Hokanson CE-4 strain gauge and photoplethysmograph was used to obtain toe pressure. A suitably sized cuff was wrapped around the proximal phalanx of the hallux. Once the best signal was acquired, the cuff was inflated until the signal disappeared, after which the cuff was slowly deflated until the signal reappeared (Application note: 85315, Hokanson D. E., Inc. Bellevue; WA, USA.). This was taken as the toe systolic pressure. Settings of equipment for all vascular assessment procedures were standardized. All measurements were carried out by the same experimenter. Each measurement was done three times, with the mean used for analysis.

### Radiographic evaluation

Measurements from standardized static weight-bearing foot radiographs have been shown to be an objective and reliable way of assessing both bony structure and soft tissue dimensions [38-42]. Weight-bearing anterior-posterior and lateral radiographs were taken with the subject standing on a platform with the central beam for the lateral view (55 kV, 8 mAs) directed horizontally at the plantar aspect of the base of the first metatarsal and the first cuneiform from a distance of 100 cm. For the anterior-posterior view (55 kV, 12 mAs), the beam was directed at the navicular from a distance of 150 cm. This radiographic protocol was previously described by Cavanagh et al[43]. A radio-opaque marker was placed on the radiographic plate in order to judge afterwards whether scale correction was necessary.

All radiographs were taken by the same radiographer, using the same equipment and settings. Measurements were taken through line drawing and a protractor was used to measure the angles. Nine angular and six linear measurements were determined from the lateral radiographs. Five angular and thirteen linear measurements were determined from the anterior-posterior radiographs. All measurements were taken by the same experimenter.

### Statistical analysis

To meet assumptions of normality of statistical distributions peak plantar pressure scores were natural log transformed. At first, data from 186 feet were used for analysis. Repeated measures ANOVA showed no statistical significant differences between left and right feet as a general effect in the repeated measures model, nor specified for the peak pressures scores (i.e. the interactions of peak pressure with left or right side), nor specified for regional peak pressure (i.e. the interactions of regional peak pressure with left or right side). Therefore it was legitimate in the eventual data analysis to average scores over left and right sides.

Next, stepwise regression analysis modelling was performed to determine which of the clinical or radiological measures will explain how much of the variance in averaged local barefoot plantar peak pressure, in each of the six forefoot regions and the whole forefoot separately. At first, all potential predictors of each subsets (clinical or radiological) were entered into the regression model through 'forward selection' and the resulting model with predictors having only statistical significant effects were noted. In the second step, all potential predictors are entered simultaneously and 'backward elimination' was applied until a model was found with predictors having statistical significant effects only. Finally, a cross-section of both resulting models was made using predictors having statistically significant effects in both models and this cross-section of predictors is 'force-entered' into the final regression model[44,45]. Comprehensive models are assembled from the significant effects of both clinical or radiological predictors following the same three-step procedure. Previously found results from literature were compared with results from our procedure. All regression analysis models were performed using list wise deletion of missing cases. To prevent type I error as much as possible in the multiple use of regression analysis, a Bonferroni correction was applied through the division by the number of plantar regions. Therefore, an alpha level of 0.01 was chosen to judge statistical significance.

To evaluate the prediction models for each individual patient the difference between the predicted value and the actual value was plotted against their mean. This method is also known as residual analysis. In the clinical literature this method is recognized as Bland-Altman analysis, which allows one to judge the agreement between two different measurement techniques[46]. Categorical data were analysed with Chi-square statistics. All data were analysed with SPSS 13.0^® ^(SPSS Inc., Chicago, USA).

## Results

### Differences between feet with and without neuropathy

#### Plantar pressure

Forefoot pressures were significant higher in patients with neuropathy, compared to patients without neuropathy for the whole forefoot, the MT-1 region and the MT-5 region (respectively 138 kPa, 173 kPa and 88 kPa higher: mean difference), table 2.

**Table 2:** Barefoot plantar peak pressure (kPa)

	**PNP- **(106 feet)			**PNP+ **(80 feet)		
	**Mean**	**SD**	**Min- Max**	**Mean**	**SD**	**Min- Max**

**Whole forefoot	551	226	234–1218	689	279	288–1262
Big toe	455	264	81–1180	405	257	24–1070
**MT-1	308	138	90–801	481	313	132–1260
MT-2	475	226	160–1210	551	271	199–1220
MT-3	429	168	187–1120	462	202	152–1000
MT-4	288	102	140–660	311	149	125–1020
*MT-5	224	147	70–782	312	238	66–1060

PNP versus PNP + *p < 0.05 & **p < 0.005

#### Clinical measurements

There were no were statistically significant differences between extremities with and without neuropathy with respect to the ankle-brachial index, toe pressure and passive dorsal flexion range of the ankle joint (p-values ≥ .126).

The range of dorsal flexion motion of the MT-1 joint was statistically significantly greater in feet without neuropathy compared to neuropathic feet: 15 degrees. The hallux valgus angle measured with a goniometer was statistically significantly greater in diabetic feet: 5.3 degrees (table 3).

**Table 3:** Clinical measurements

	**PNP-**			**PNP+**		
	**Mean**	**SD**	**Min-Max**	**Mean**	**SD**	**Min-Max**

**Vibration Perception Threshold	13,78	5,05	4,8–25	43,55	8,82	25,2–50
**Valk score	2,1	3,1	0–15	12,9	7,3	0–24
Ankle-Arm index	1,06	0,19	0,52–1,83	1,08	0,28	0,63–2,3
Toe pressure	109,5	29,8	47,5–200	113,8	36,9	44–255
Dorsal flexion ankle joint	10	6,4	-5–25	9	6,9	-5–25
**Dorsal flexion motion MT-1 joint	93	16,8	45–150	78	16,0	40–110
**Hallux valgus angle	11,1	5,6	0–25	16,4	11,4	0–50

**PNP- versus PNP+ p < 0.005

#### Callus formation and toe deformity

**Figure 1:**
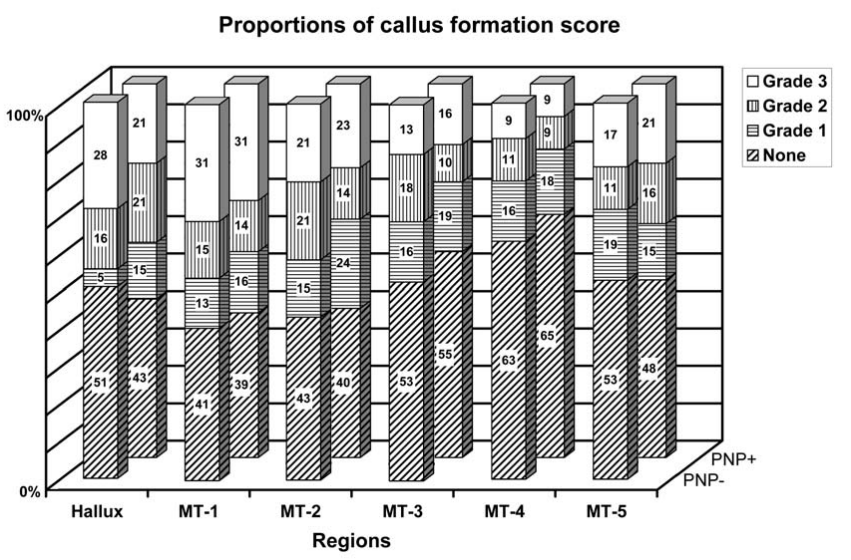
**Proportions of callus formation score, as a percentage, for patients with and without neuropathy**. None = no thickening of keratin layer, Grade 1 = Distinct area with minimal thickening of keratin layer, Grade 2 = moderate thickening of keratin layer, Grade 3 = marked thickening of keratin layer. MT = metatarsal, PNP - = without neuropathy, PNP + = with neuropathy.

**Figure 2:**
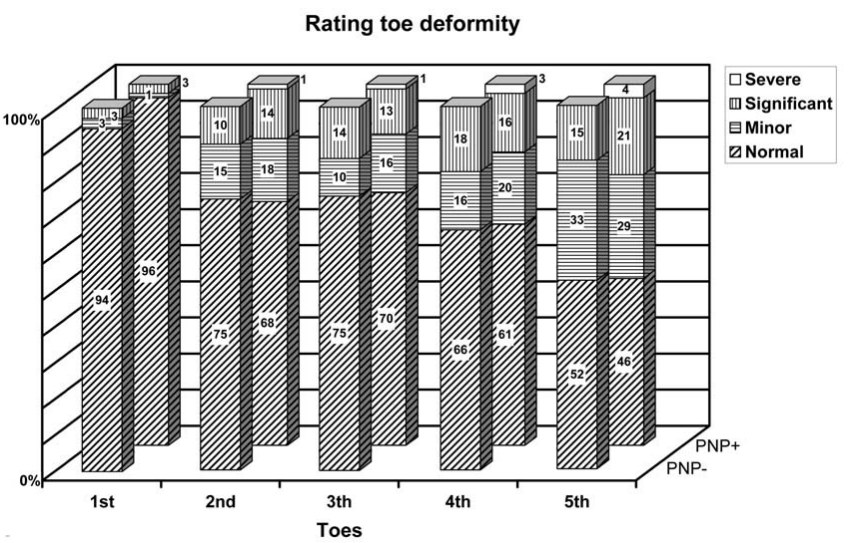
**Proportions of toe deformity ratings, as a percentage, for patients with and without neuropathy**. Normal = no malalignment whereas minor, significant and severe are respectively defined as less, the same or more deformed than the definitions described by Myerson and Shereff.

The distributions of callus formation and toe deformity grades were similar for both feet with and without neuropathy (figures 1 and 2). Most important callus formation was found under the hallux and the head of MT-1, while the least callus formation was found under the lateral side of the forefoot: MT-3 to MT-5.

For both groups the grade of toe deformity increased from the first to the fifth toe. No statistically significant differences for callus formation and toe deformity were found (p-values > .056), except that neuropathic feet had a slightly higher grade of deformity of the 4^th ^and 5^th ^toe (p-values < .042).

#### Calcaneal alignment

The proportions for classification of the calcaneal resting position for feet with and without neuropathy was: 'neutral' 47.2 percent *versus *51.2 percent; 'valgus' 46.2 percent *versus *47.5 percent and 'varus' 6.6 percent *versus *1.3 percent, respectively.

#### Arch height

The proportions of arch height classification for patients with and without neuropathy were: 'no arch' 6.6 percent *versus *13.8 percent; 'lowered arch' 42.5 percent *versus *31.3 percent; 'normal arch' 17.9 percent *versus *21.1 percent; 'elevated arch' 26.4 percent *versus *32.5 percent and 'excessively elevated arch' 6.6 percent *versus *1.3 percent.

No statistically significant differences between neuropathic feet and feet without neuropathy for both calcaneal resting position and arch height were found (p > .062).

#### Radiographic evaluation

The statistically significant differences for patients with neuropathy compared to patients without neuropathy listed as radiographic measures in millimetres (mm) and degrees (deg) were: MT-5 base height -0.6 mm, navicular height -3.7 mm, inferior calcaneal inclination -2.9 deg, Chopart's joint angle -1.4 deg, Lisfranc's joint angle +2.0 deg, MT-5 inclination -1.5 deg, MT-1 inclination -1.6 deg, Talar inclination +3.0 deg, 1st PIP joint inclination -2.1 deg, hallux valgus +2.4 deg, MT-2 thickness +0.4 mm and MT-3 thickness +0.4 mm (table 4).

**Table 4:** Radiographic measurements

	**PNP-**			**PNP+**		
**Lateral radiograph**	**Mean**	**SD**	**Min-Max**	**Mean**	**SD**	**Min-Max**

Sesamoid height (mm)	4.6	2.35	0.5–14	4.2	2.63	0–15
MT-5 head height (mm)	8.3	2.21	3.5–18	7.7	2.23	2–13.5
*MT-5 base height (mm)	15.4	3.91	4–26	14.1	4.50	5–24
**Navicular height (mm)	40.8	8.66	10–60	37.1	8.78	12.5–53
Calcaneal height (mm)	9.5	2.91	3–20	9.6	3.05	3–17
**Inferior calcaneal inclination (deg)	23.4	5.18	11–36.5	20.5	5.78	3–31
superior calcaneal inclination (deg)	21.7	5.52	8.5–35	20.7	7.12	2–35.5
*Chopart's joint angle (deg)	63.3	4.43	47–75.5	61.9	4.83	46–71.5
Navicular 1st cuneiform angle (deg)	64.0	5.20	49.5–76.5	64.7	4.47	56–77.5
**Lisfranc's joint angle (deg)	63.3	4.03	52.5–74	65.3	5.09	56–86
**MT-5 inclination (deg)	12.8	3.97	3.5–23.5	11.3	3.63	1–18.5
*MT-1 inclination (deg)	23.7	3.70	11.5–33	22.1	4.23	6.5–30
**Talar inclination (deg)	25.0	4.86	12.5–43	28.0	6.22	16–49.5
*1st PIP joint inclination (deg)	10.9	4.82	-3.5–25	8.8	7.01	-14–31.5
						
**Anterior-posterior radiograph**	**Mean**	**SD**	**Min-Max**	**Mean**	**SD**	**Min-Max**
*Hallux valgus (deg)	14.3	5.22	4–29	16.7	10.96	1.5–56.5
Intermetatarsal 1–5 angle (deg)	26.1	3.25	19–36.5	25.4	5.65	9.5–41
Intermetatarsal 1–2 angle (deg)	10.9	2.66	6–25	10.4	3.40	5–22
MTP-1 angle (deg)	14.3	5.22	4–29	16.7	10.96	1.5–56.5
Interphalangeal 1 angle (deg)	9.7	5.06	0–23.5	10.3	5.99	-10–26
Medial sesamoid X deviation (mm)	2.3	1.97	-2.5–7	1.8	3.32	-8–8.5
Medial sesamoid Y deviation (mm)	12.3	2.45	6–21	12.2	2.40	5.5–17.5
Lateral sesamoid X deviation (mm)	9.2	1.67	3.5–16	9.7	1.90	5–13.5
Lateral sesamoid Y deviation (mm)	15.4	2.45	7.5–21.5	16.0	3.11	8–24
Morton's index (mm)	1.5	2.94	-6–6.5	1.2	3.80	-5.5–17
MT-1 thickness (mm)	13.9	1.79	10.5–19.5	14.0	1.71	11–19
*MT-2 thickness (mm)	7.3	0.85	5.5–10	7.7	1.07	6–12.5
*MT- 3 thickness (mm)	6.4	0.71	5–8	6.8	1.11	5–12
MT-1 lenght (mm)	65.2	5.04	56–83.5	66.6	4.76	55–76.5
MT- 2 lenght (mm)	77.4	4.96	67.5–95.5	78.3	5.36	64.5–88
MT- 3 lenght (mm)	75.1	4.96	64.5–93	75.9	5.74	64–90
MT-4 lenght (mm)	73.3	4.93	61–88	74.3	5.32	54–83
MT- 5 lenght (mm)	72.9	5.24	61–84.5	73.3	5.02	57–82

PNP- versus PNP+ *p < 0.05 & **p < 0.005, PIP = proximal interphalangeal, deg = degree

### Prediction of barefoot plantar peak pressure

#### Regression models

With a maximum of 5 predictors, the regression models with clinically independent variables explained up to 34 percent of variance of local peak pressures (table 5). The regression models with clinical variables explained more variance for the medial forefoot and the hallux than for the lateral forefoot. Callus formation was a relevant clinical predictor for all regions and the 'Valk score' for two regions and the whole forefoot. For the hallux region, the best model with 24 percent of explained variance of peak pressure was also achieved with clinical variables.

**Table 5:** 

	**Clinical predictors**						**Radiological predictors**						**Comprehensive model**					
**Region**	**Variable**	***R***	***R*^2^**	β	**SE**	**Beta**	**Variable**	***R***	***R*^2^**	β	**SE**	**Beta**	**Variable**	***R***	***R*^2^**	β	**SE**	**Beta**

**Fore foot**	model	0,37^†^	**0,14**				model	0,42^†^	**0,18**				model	0,51^†^	**0,26**			
	Intercept			3,863^†^	0,038		Intercept			4,039^†^	0,200		Intercept			3,605^†^	0,065	
	Valk score			0,019^†^	0,004	0,35	MT-1 phalan angle			0,019^†^	0,005	0,40	Valk score			0,028^†^	0,006	0,31
	Toe deformity sum			0,017^†^	0,076	0,29	Sesamoid height			-0,043*	0,015	-0,20	Callus sum			0,018*	0,006	0,21
							Med sesam Y dev			-0,053*	0,015	-0,24	Moton's index			0,027*	0,008	0,22
													Deviation hallux rx			0,010*	0,003	0,20

**Hallux**	model	0,49^†^	**0,24**				model	0,38^†^	**0,14**				model	0.38^†^	**0.14**			
	Intercept			3,886^†^	0,149		Intercept			-0,128^†^	0,708		Intercept			-0,175^†^	0,704	
	Callus formation			0,176^†^	0,036	0,32	Nav 1st cuneiform			0,040^†^	0,010	0,28	Nav 1st cuneiform			0,040^†^	0,010	0,28
	Calcaneus varus			-0,266*	0,094	-0,19	MT-5 inclination			0,033*	0,013	0,18	MT-5 inclination			0,033*	0,013	0,18
	Dorsal flex ankle			-0,026*	0,008	0,22	Lat sesam Y dev			0,046*	0,018	0,18	Med sesam Y dev			0,046*	0,018	0,18
	Height medial arch			-0,151^†^	0,043	-0,23												

**MT-1**	model	0,55^†^	**0,30**				model	0,42^†^	**0,19**				model	0,58^†^	**0,34**			
	Intercept			3,102^†^	0,058		Intercept			4,039^†^	0,200		Intercept			3,572^†^	0,170	
	Valk score			0,025^†^	0,004	0,36	Sesamoid height			-0,043^†^	0,015	-0,20	Valk score			0,024^†^	0,004	0,35
	1st toe deformity			0,271*	0,095	0,18	Med sesam Y dev			-0,053*	0,015	-0,24	Callus formation			0,133^†^	0,025	0,33
	Callus formation			0,135^†^	0,026	0,33	MT-1 phalan angle			0,019*	0,005	-0,29	Deviation hallux rx			0,012*	0,004	0,20
													Med sesam Y dev			-0,051^†^	0,014	-0,24

**MT-2**	model	0,46^†^	**0,21**				Model	0,40^†^	**0,16**				model	0,50^†^	**0,25**			
	Intercept			3,483^†^	0,064		Intercept			2,656^†^	0,260		Intercept			2,607^†^	0,237	
	Callus formation			0,126^†^	0,026	0,33	Devi hallux rx			0,016^†^	0,004	0,29	Callus formation			0,134^†^	0,025	0,35
	2nd toe deformity			0,111*	0,043	0,17	MT-2 thickness			0,118^†^	0,032	0,25	MT-2 thickness			0,133^†^	0,031	0,29
	Valk			0,005*	0,002	0,19	Moton's index			0,027*	0,009	0,20	2nd toe deformity			0,145*	0,042	0,23

**MT-3**	model	0,35^†^	**0,12**				Model	0,29^†^	**0,09**				model	0.40^†^	**0.16**			
	Intercept			3,251^†^	0,137		Intercept			3,531^†^	0,059		Intercept			3,220^†^	0,136	
	Callus formation			0,102^†^	0,024	0,30	Moton's index Dev			0,025*	0,008	0,22	Callus formation			0,098^†^	0,023	0,29
	Body Mass Index			0,013*	0,005	0,20	hallux			0,010*	0,003	0,22	Body Mass Index			0,013*	0,004	0,18
													Moton's index			0,021*	0,008	0,20

**MT-4**	model	0,26^†^	**0,07**				Model	0,25^†^	**0,06**				model	0,35^†^	**0,13**			
	Intercept			3,263^†^	0,030		Intercept			2,030^†^	0,371		Intercept			2,026^†^	0,359	
	Callus formation			0,095^†^	0,026	0,26	MT-4 length			0,018^†^	0,005	0,25	Callus formation			0,091^†^	0,025	0,25
													MT-4 length			0,017^†^	0,005	0,24

**MT-5**	model	0,40^†^	**0,16**				Model	0,47^†^	**0,22**				model	0,54^†^	**0,29**			
	Intercept			2,833^†^	0,056					1,448*	0,596		Intercept			1,933*	0,575	
	Callus formation			0,215^†^	0,036	0,40	MT-5 length			0,033^†^	0,008	0,27	Callus formation			0,181^†^	0,034	0,34
							Moton's index			-0,035*	0,013	-0,18	MT-5 length			0,023*	0,018	0,19
							MT-5 head height			-0,096^†^	0,019	-0,34	MT-5 head height			-0,093^†^	0,008	-0,33

* = p < .01, ^† ^= p < .0001, MT = metatarsal, PIP = proximal interphalangeal, phalanx = phalangeal, nav = navicular, sum = summation of scores, med = medial, lat = lateral, dev = deviation

The regression model with radiological variables for the forefoot peak pressure with 4 predictors explained 18 percent of the variance of plantar pressure. The model for the MT-5 region explained no more than 22 percent of the variance, which was the best result with radiological variables compared to the models for the other regions.

For most regions, the comprehensive model i.e. the combination of clinical and radiological predictors, resulted in the largest explained variance: 26% (forefoot), 34% (MT-1 region), 25% (MT-2 region), 16% (MT-3 region), 13% (MT-4 region) and 29% (MT-5 region), table 5.

#### Previously proposed regression models

We used the same radiological measures in the present study as in previous studies by Morag and Cavanagh (1999)[47]. The model for the hallux region, yielded an *r *of .061 and an *r*^2 ^of .004, SE 0.71 (overall p-value .877).

The models for the MT-1 region proposed by Cavanagh et al (1997)[38], resulted in *r *.27, *r*^2 ^.075, SE 0.51 (p = .007) and Morag and Cavanagh (1999)[47], *r *.31, *r*^2 ^.10, SE 0.51 (p = .002). The only statistically significant beta, also known as beta weight or standardized regression coefficient, in the MT-1 model was 'sesamoid height', i.e. soft tissue thickness under the first metatarsalphalangeal joint.

We also evaluated proposed regression models by Mueller et al.(2003)[48], Ahroni et al. (1999)[49] and Payne et al. (2002)[50] with similar variables from our dataset for the plantar regions concerned. No statistically significant regression models were found for the hallux region.

The models proposed by Mueller et al. for MT-1 (hallux valgus angle, Morton's index, body weight) to MT-3 (hammer toe deformity, soft tissue stiffness, calcaneal inclination) and MT-5 (hammer toe deformity, Morton's index) were statistically significant: MT-1 region *r *.32, *r*^2 ^.105 SE 0.50 (p = .001); MT-2 region *r *.32, *r*^2 ^.103, SE 0.43 (p = .001); MT-3 region *r *.22, *r*^2 ^.046, SE 0.38, (p = .013) and MT-5 region R .29, *r*^2 ^.072, SE 0.61 (p = .004). This was also true for the model for prediction of plantar pressure under the forefoot by Ahroni et al. (body weight, insulin use) and the model for the MT-1 region suggested by Payne et al. (MTP-1 range of motion, Michigan Neuropathy Score): *r *.46, *r*^2 ^.212, SE 0.19 (p = .001) and *r *.36, *r*^2 ^.131, SE 0.49 (p = .001), respectively.

#### Bland-Altman analysis

**Figure 3:**
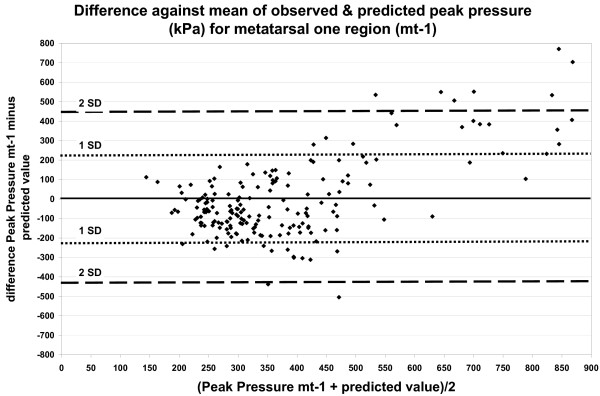
**The difference between the observed and predicted peak pressure plotted against the mean (kPa), based on the comprehensive regression model for MT-1 region**. Comprehensive model (*r*^2 ^34%). Independent variables: Valk score, Deviation hallux x-ray, Callus formation, Medial sesamoid Y-axis deviation.

**Figure 4:**
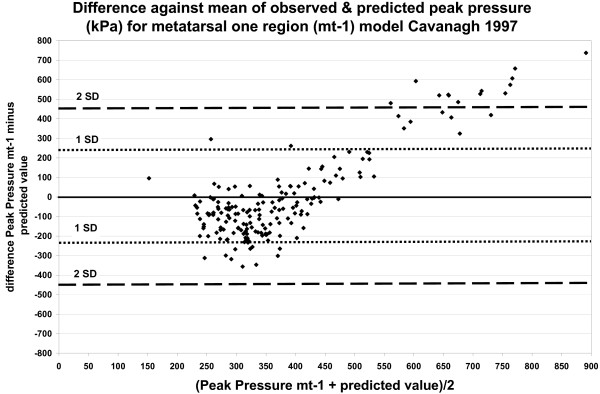
**The difference between the observed and predicted peak pressure plotted against the mean (kPa), based on the regression model for MT-1 region stated by Cavanagh et al. (1997)**. Model with radiological variables (*r*^2 ^38%). Independent variables: 1st Metatarsal length, Sesamoid height, 1st Metatarsal-phalangeal angle.

Figures 3 and 4 show the difference between the observed and predicted peak pressure plotted against their mean for the MT-1 region. Figure 3 shows the results based on the comprehensive regression model and figure 4 shows the results based on the regression model proposed by Cavanagh et al. (1997)[38]. Figures 3 and 4, mean values between 200 kPa and 450 kPa, show both positive and negative differences of circa 200 kPa, while the difference between the observed and predicted peak pressure increases at higher values.

The dotted lines in figures 3 and 4, indicate the 65 percent range of differences (1 SD) and the range for 95 percent of differences (2 SD).

## Discussion

We explored new and previously suggested relationships between clinical measurements, data from radiographs and barefoot plantar pressure in patients with diabetes. The primary aim of the present study was to find a minimal combination of measures which could indicate locations with elevated plantar pressure as an alternative for quantitative plantar measurement.

### Results from clinical examination and radiographic evaluation

Consistent with what is reported in the literature, higher peak pressures were measured under the neuropathic forefoot than those under the forefoot without neuropathy[51]. The greatest difference was measured in the MT-1 region. The dorsal flexion motion of the metarsalphalangeal joint of the first toe was less while the hallux valgus angle was greater in neuropathic feet compared to feet without neuropathy. Both observations were previously reported in the literature and are positively correlated with plantar pressure[38,43,52,53].

The distributions of callus formation and toe deformity grades were similar for feet with and without neuropathy. Neuropathic feet had a slightly higher grade of deformity of the 4^th ^and 5^th ^toe. Results from the regression analysis showed that toe deformities were weakly associated with peak pressures in their pertaining forefoot regions. There were no statistically significant differences in calcaneal resting position and arch height between feet with and without neuropathy. Callosities, toe deformity, calcaneal alignment and arch height were previously identified as distinct features in high risk diabetic patients with neuropathy[1,49,54,55]. The severity of these clinical symptoms in the patients of this study are relatively mild. This could be the explanation for the minor differences between feet with and without neuropathy.

Relatively many differences were found for measurements on lateral radiographs compared to anterior-posterior radiographs. The soft tissue thickness under the head of MT-5 is greater in patients without neuropathy, which could be an explanation for the lower peak pressure in this region compared to patients with neuropathy. A similar relationship between soft tissue thickness and peak pressure exists for MT-1 and was reported in previous studies[5,38,56].

Various radiological parameters indicate that neuropathic feet have a lower arch structure than feet without neuropathy. Some parameters obtained from lateral and anterior-posterior radiographs indicate similar classifications found through clinical examination, such as arch height and hallux alignment, although no statistically significant differences were found.

### Regression models

In general, the regression models with selected clinical variables explained more variance in plantar pressure data than the models with only radiological measures, suggesting that clinical examination tells us more about plantar pressure than information from radiographs.

For most regions, the prediction of plantar pressure improved with the combination of radiological and clinical data. The results show that clinical and radiological measurements, either independently or in combination, could explain only about 34 percent of the variance in local bare foot peak pressure. In other words, with the models constructed we have only 34 percent of useful information from the clinical and radiological measurements to make an prediction about the actual bare foot peak pressure.

The Bland-Altman method was used for a visual impression of the individual difference between the predicted and the actual pressure values. We evaluated the comprehensive regression model for the MT-1 region. The scattered data along the wide interval of the horizontal axis in figure 3 show a great variation. The considerable discrepancy between the predicted and the actual peak pressure indicating the inadequacy of the model, especially with regard to the underestimation of high plantar pressure values, i.e. > 700 kPa, for which an accurate estimation is highly important.

In general, the previously reported regression models with independent variables from clinical examination showed similar results to our study. Ahroni et al. (1999) claimed that high in-shoe plantar pressure in diabetic subjects (1,017 feet) could be predicted through the use of clinical variables, such as body mass, insulin use, Caucasian race, male sex, plantar callus and diabetes duration[49]. Only 12 percent of the variation was explained. Although Ahroni in his study found body mass to be identified as the most important factor, Cavanagh et al. (1991) found that body mass was a poor predictor[57].

Various predictive models relating plantar peak pressure and structural variables obtained through imaging techniques have been suggested)[38,47-49,58-62]. Cavanagh et al. presented a model which explained approximately 40 percent of the variance in forefoot pressure during walking with 3 to 4 static structural measurements from foot radiographs of healthy subjects[38]. While we found a similar result, 34 percent of explained variance is not sufficient for prediction of plantar pressure.

#### Prediction models with the same or similar parameters applied to the present data set

Various regression models with demographically, clinically and radiologically independent variables have been suggested in the literature to estimate local plantar pressure in patients with diabetes. Comparison of these models is difficult. Various methodological aspects are different such as the population characteristics, the choice of the plantar pressure parameters and how the data of the independent variables were obtained. Despite these differences, we constructed regression models with the same or similar parameters and applied these models to our data set.

We used radiological measures identical to those used by Morag and Cavanagh (1999)[47] for the prediction of barefoot peak pressure, but we could reach only 13 percent explained variance. Cavanagh et al. (1997)[38] suggested four radiological measures for peak pressure in the MT-1 region. This model was also evaluated with our data and analysed with the Bland-Altman method (figure 4). Similarly modest results as for our own comprehensive MT-1 model (figure 3) were found. Twenty-one percent was the highest explained variance found, with the regression models formulated by Mueller et al.(2003)[48], Ahroni et al. (1999)[49] and Payne et al. (2002)[50], which implies that these models are not useful when applied to the data of the present study.

This study was directed from a practical and ecological research perspective, which means that the patients were mostly screened according through common practice procedures: e.g. goniometry, blood pressure measurements, etc. According to scientific criteria, these common practice procedures often have inferior clinimetric qualities and are likely to contribute to inherent variability. Maybe other clinical or radiological measurements are less prone to inherent variability and may be stronger predictors for local peak pressure. We studied diabetic patients without foot complications such as severe deformities and gait abnormalities. Some of the results might well be different in patients with these complications of diabetes. Nevertheless, we think it is important to identify patients with elevated plantar pressures in their early stage of disease in order to take appropriate preventive measures. The screening of these kind of patients is typically done in peripheral care centres where there is no radiological facility or plantar pressures equipment are available.

Clinical signs are potentially more cost-effective than sophisticated diagnostic tests which are less feasible in community settings. However, the results form this study confirm the conclusions of a previous study which showed that identification of locations with elevated plantar pressure is not possible through physical examination[63]. The use of radiological measures does not provide a better prediction of local barefoot peak pressure. There is a clear need for further research to address these clinical uncertainties. The use of quantitative plantar pressure measurement is therefore advocated for diabetic foot care.

## Conclusion

Peak pressures were higher in patients with neuropathy than in patients without neuropathy, although feet were quite similar as far as clinical and radiological evaluations is concerned. At best, clinical and radiological measurements could explain about 34 percent of the variance in local barefoot peak pressure in this population of diabetic patients. The prediction models constructed with regression modeling were not useful in clinical practice, because of considerable discrepancies between the predicted and the actual peak pressure values. Identification of elevated plantar pressure without equipment for quantification of plantar pressure is inadequate. This points toward the merit of quantitative plantar pressure measurement for diabetic foot screening.

## Pre-publication history

The pre-publication history for this paper can be accessed here:


